# Identification of Novel MAGE-G1-Interacting Partners in Retinoic Acid-Induced P19 Neuronal Differentiation Using SILAC-Based Proteomics

**DOI:** 10.1038/srep44699

**Published:** 2017-04-04

**Authors:** Yong Liu, Yujian Chen, Shide Lin, Shuguang Yang, Shaojun Liu

**Affiliations:** 1 State Key Laboratory of Proteomics, Department of Neurobiology, Institute of Basic Medical Sciences, Beijing, 100850, China

## Abstract

MAGE-G1 is a protein plays role in the early process of neurogenesis. However, the fundamental roles MAGE-G1 played in neurogenesis have not yet been completely understood. Finding the partners MAGE-G1 interacting with will surely contribute to the function study of MAGE-G1. In this study, using Stable Isotope Labeling by Amino acids in Cell culture-immunoprecipitation quantitative proteomics, we screened the interacting proteins of MAGE-G1 during retinoic acid -induced neuronal differentiation of P19 cells and firstly found that FSCN1 and VIME were potential novel MAGE-G1-interacting proteins. Then, the interaction between overexpressed MAGE-G1 and FSCN1 or VIME was validated by GST-pull down assay in bacteria and by co-immunoprecipitation assay in COS7 cells. Endogenous co-immunoprecipitation assay further confirmed that MAGE-G1 interacted with FSCN1 or VIME in P19 cells after a 6-day retinoic acid-induced neuronal differentiation. Those results provide a functional linkage between MAGE-G1 and FSCN1 or VIME and may facilitate a better understanding of the fundamental aspects of MAGE-G1 during neurogenesis.

The melanoma antigen (MAGE) family comprises 30 proteins, and is divided into two classes based on their expression patterns, the type I cancer-testis antigen and the type II ubiquitous MAGEs^1^,^2^. Both type I and type II MAGE proteins share a conserved domain known as the MAGE homology domain (MHD). Based on similarities in the MHD sequences and molecular sizes, the type II MAGE proteins can be divided further into two subgroups, Necdin subgroup and MAGE-D subgroup. Necdin subgroup consists of relatively short proteins (<350 amino acid residues) whose MHD sequences are more homologous to that of Necdin. This subgroup includes Necdin, MAGE-F1, MAGE-G1, and MAGE-H1 in mammals. MAGE-D subgroup consists of larger proteins (>650 amino acid residues), including MAGE-D1/NRAGE/Dlxin-1, MAGE-D2, MAGE-D3/Trophinin/Magphinin, MAGE-E1/MAGE-D4, and MAGE-L2^2^,^3^.

As one of *Necdin* homologous genes, *Mage*-*g1* gene (also designated *Necdin*-*like 2*) has been mapped to proximal chromosome 15q^4^. The proximal region of human chromosomal 15q is subject to genomic imprinting and implicated in various human neurological and mental disorders including Prader Willi Syndrome, Angelman syndrome, autism, epilepsy, and schizophrenia^5^. It is demonstrated that MAGE-G1 has some characteristics similar to those of Necdin, such as growth suppression and interactions with E2F transcription factor 1 (E2F1) and p75 neurotrophin receptor (p75NTR)^6^. MAGE-G1 is a member of type II MAGE proteins of which some member are key elements in neurogenesis, such as MAGE-D1, MAGE-L2^2^,^7^. Our previous studies found that the expression of MAGE-G1 protein was increased with the differentiation of P19^8^. Those findings suggest that the MAGE-G1 may be involved in brain development, and its abnormalities may cause some neurodevelopmental diseases. However, the biochemical and functional features of MAGE-G1in brain development and neurogenesis remain unknown.

Identification of relevant interacting proteins is an essential step in investigating protein functions. A number of techniques are used to screen unknown interacting proteins, which include the yeast two-hybrid system, pull-down assays, as well as tandem affinity purification (TAP)^9^,^10^. However, those techniques suffer from high false positive and false negative rates, because the assay is usually performed under non-physiological conditions and the posttranslational dynamics are not taken into account. Stable Isotope Labeling by Amino acids in Cell culture (SILAC)-immunoprecipitation quantitative proteomics provides a useful tool to overcome the disadvantages mentioned above^11^. Using this method to screen interacting proteins, specific partners appear as isotopically heavy, while non-specific interaction partners appear as a mixture of isotopically light and heavy at a 1:1 ratio. The SILAC-immunoprecipitation quantitative proteomics has some significant advantages in identification of interaction partners. For example, the cell localization and post-translational modifications are not perturbed; as a quantitative approach, it allows the user to readily distinguish non-specifically interacting proteins from host factors that bind specifically; this method enables the identification of not only direct interacting partners but also low affinity or indirect interacting partners^12^,^13^.

In this study, we identified interacting partners of MAGE-G1 during retinoic acid (RA)-induced neuronal differentiation of P19 cells using SILAC-immunoprecipitation quantitative proteomics, and found that FSCN1 and VIME were potential novel MAGE-G1-interacting proteins. The interactions were further validated by both exogenous and endogenous co-immunoprecipitation assay.

## Results

### Screening Potential MAGE-G1-interacting Proteins by SILAC-Immunoprecipitation Quantitative Proteomics Approach

**Figure 1:**
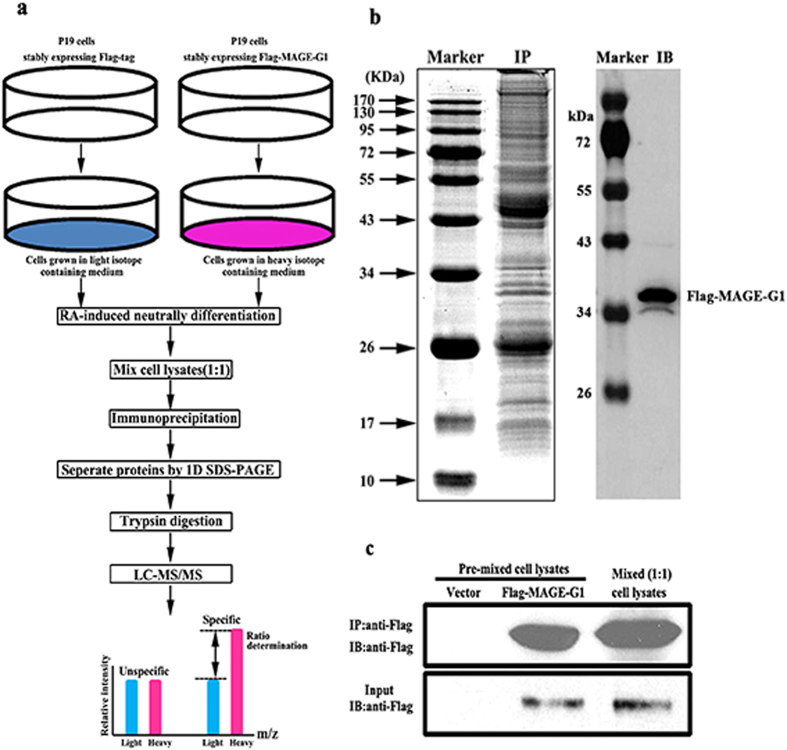
Isolation of MAGE-G1-interacting complex by immunoprecipitation. (**a**) Strategy to identify MAGE-G1-interacting partners during RA-induced neuronal differentiation of P19 cells. P19 cells stably expressing Flag-MAGE-G1 were maintained in “heavy” medium, while control cells stably expressing Flag-tag were grown in “light” medium. After 6-day treatment with RA, the whole cell lysates extracted from each cell pool were mixed 1:1 based on the total protein mass. The MAGE-G1-interacting complex was purified using anti-Flag beads followed by SDS-PAGE separation, in-gel trypsin digestion, and LC–MS/MS analysis. (**b**) Isolation of the MAGE-G1 complex by immunoprecipitation. Left line, the immunoprecipitated proteins were separated by SDS-PAGE and stained with coomassie brilliant blue. *IP* immunoprecipitates. Right line, Flag-Mage-G1 protein was detected by anti-Flag antibody. *IP* immunoprecipitation; *IB* immunoblotting. (**c**) Identification of Flag-MAGE-G1 immunoprecipitated by anti-Flag beads in pre- or mixed cell lysates (1:1) by immunoblotting. *IP* immunoprecipitation; *IB* immunoblotting.

A P19 cell line which stably expressed the Flag-tagged mouse MAGE-G1 was generated, and then the specific MAGE-G1 interactome formed in P19 cells during RA-induced neuronal differentiation was screened using the SILAC-based quantitative proteomic approach. Briefly (Fig. 1a), the P19 cells expressing Flag-MAGE-G1 were grown in the “heavy” medium containing ^13^C_6_ L-lysine, whereas the control cells (transfected with empty vector) were maintained in “light” medium containing ^12^C_6_ L-lysine. P19 cells were treated with RA for 6 days to induce neuronal differentiation, and then the proteins were extracted from each group and mixed in a ratio of 1:1 based on the total protein mass. Anti-Flag beads were added to immunoprecipitate the MAGE-G1 interacting complex followed by SDS-PAGE separation (Fig. 1b), in-gel trypsin digestion, and LC–MS/MS analysis. Immunoblotting was conducted to confirm whether Flag-MAGE-G1 was immunoprecipitated from cell lysates (Fig. 1c).

### SILAC Analysis Discriminates the Specific Binders from the Unspecific

**Figure 2:**
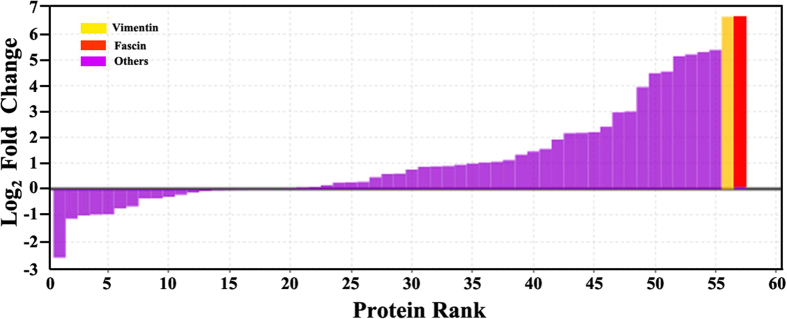
SILAC analysis discriminates specific from unspecific binders. The bars represent the Log 2 base (Log_2_) value of fold change heavy-to-light for each identified protein. Protein rank refers to the numbers of identified proteins. Protein FSCN1 and VIME are considered specific and other proteins (purple bars) unspecific.

**Figure 3:**
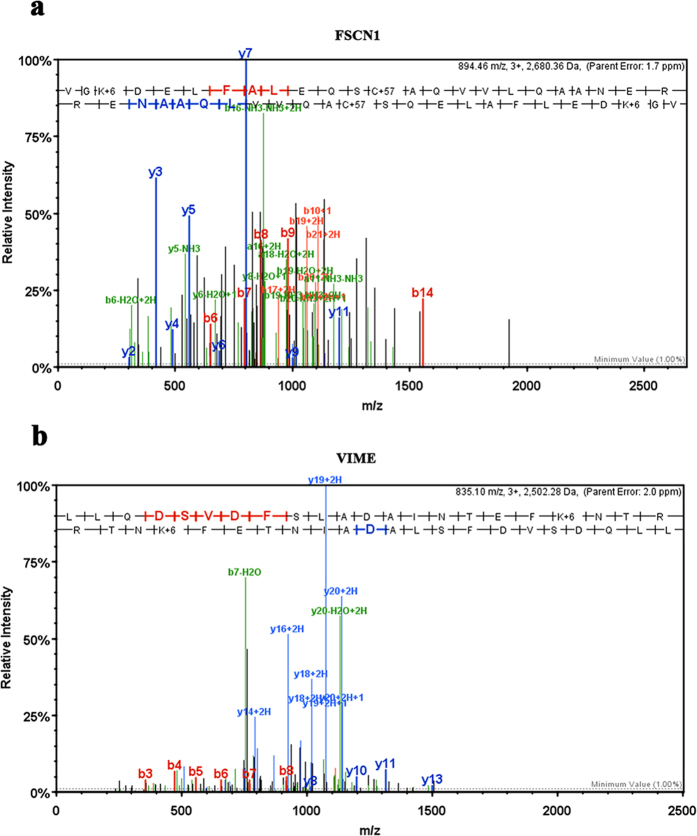
MS/MS fragmentation spectrum identifies the peptide of MAGE-G1-interacted proteins. (**a**) The peptide sequence was determined from MS/MS fragmentation spectrum as (VGKDELFALEQSCAQVVLQAANER), and identified as mouse FSCN1 peptide. (**b**) The peptide sequence was determined from MS/MS fragmentation spectrum as (LLQDSVDFSLADAINTEFKNTR), and identified as mouse VIME peptide.

According to the stringent criteria for protein identification (see Materials and Methods), a total of 57 proteins were quantified with L/H ratios (Fig. 2 and Supplementary Table 1). Using significance B value (p < 0.05) as the threshold to distinguish the specific MAGE-G1-interacting proteins^14^, 27 proteins were demonstrated as having significant abundance changes (L/H ratios > 1.70 *i.e*. Log_2_ (L/H ratios) >0.7) (Table 1). However, certain highly abundant proteins, such as cytoskeletal proteins, histones, hnRNP proteins, and ribosomal proteins, were also included in the list. Based on the previous report^15^, they are in the class of “beads proteome”, *i.e*., proteins that often bind to the agarose beads where the Flag antibody is conjugated to, therefore, co-purify with the “true” interacting partners during the process of immunoprecipitation. Thus, after excluding these unspecific proteins, FSCN1 and VIME were identified as potential MAGE-G1-interacting proteins formed during RA-induced neuronal differentiation of P19 cells. Figure 3 shows the MS/MS fragmentation spectrum identifies the peptide of FSCN1 (Fig. 3a) and VIME (Fig. 3b).

### Validation of the Interaction between MAGE-G1 and FSCN1

**Figure 4:**
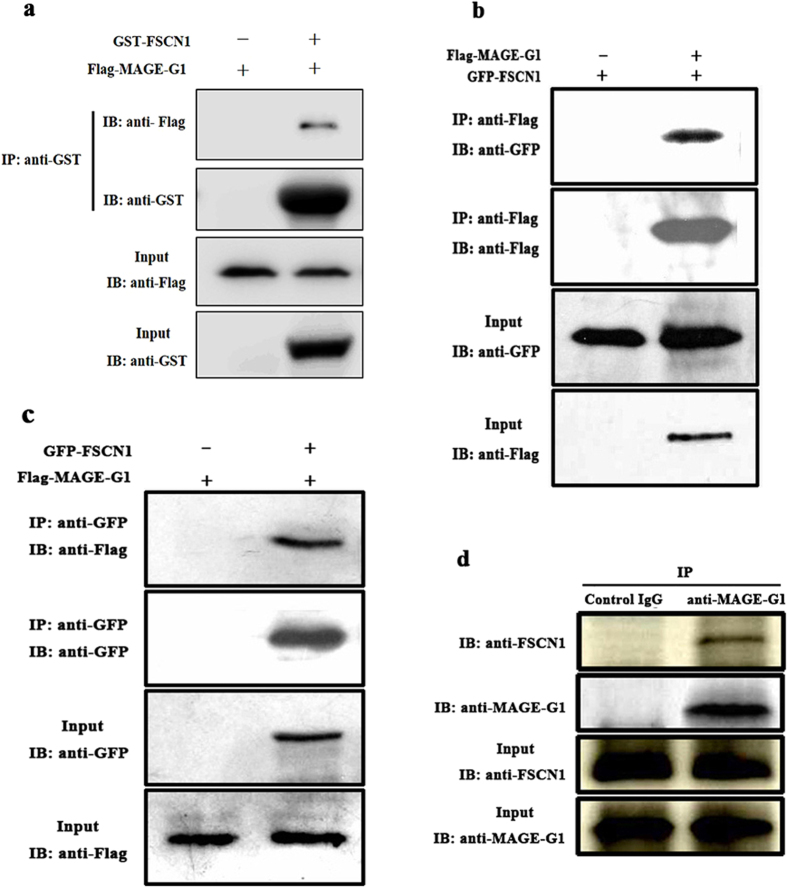
Validation of the interaction between MAGE-G1 and FSCN1 by GST pull-down and co-immunoprecipitation experiments. (**a**) GST or GST-FSCN1 proteins were expressed in *Escherichia coli* BL21 respectively and purified with Glutathione-Sepharose 4B beads and washed, then beads were incubated with Flag-MAGE-G1 expressed in HEK293T. Flag-MAGE-G1 and GST-FSCN1 were detected with indicated antibody. Full-length blots are included in a Supplementary Information. (**b**) COS-7 cells were co-transfected with either Flag-*Mage*-*g1* plus GFP-*Fscn1* or pCMV-3 × Flag empty vector plus pEGFP-*Fscn1* expression plasmids. 25 μg of whole cell protein lysate was used as input to confirm the expression of the Flag-MAGE-G1 (with anti-Flag) or GFP-FSCN1 (with anti-GFP) by immunoblotting (IB). The rest of cell lysates were incubated with anti-Flag-magnetics beads. The immunoprecipitated (IP) protein complex was resolved by SDS-PAGE and probed with antibodies against Flag or GFP. (**c**) COS-7 cells were co-transfected with either Flag-*Mage*-*g1* plus GFP-*Fscn1* or pEGFP empty vector plus Flag-*Mage*-*g1* expression plasmids. The experiment procedure was same as that mentioned above except that cell lysates were immunoprecipitaed with anti-GFP-magnetics beads. (**d**) Whole cell lysates from RA-treated P19 cells were immunoprecipitated with anti-MAGE-G1 antibody. IgG antibody was used as negative control of immunoprecipitation (IP) and 25 μg whole cell lysate was used as input. The immunoblotting (IB) were probed for the immunoprecipitated proteins with anti-FSCN1 antibody.

To investigate the interaction between MAGE-G1 and FSCN1, we detected the association of these two proteins by GST pull-down assay in bacteria and by immunoprecipitation in transfected mammalian cells. GST or GST-FSCN1expressed in *Escherichia coli* BL21 were incubated with Flag-MAGE-G1 expressed in HEK293T, and precipitated by Glutathione-Sepharose 4B beads. The result showed that Flag-MAGE-G1 was detected in the GST-FSCN1 precipitate (Fig. 4a). The expression vectors that encoded MAGE-G1 fused with a Flag-tag (pCMV-3 × Flag-*Mage*-*g1*) and *Fscn1* fused with a GFP-tag (pGFP-N1-*Fscn1*) were prepared, and then were transiently co-expressed in COS-7 cells. Empty vectors pCMV-3 × Flag and pGFP-N1 were used as control. Immunoprecipitation and immunoblotting analysis were performed on cell lysates from those transfected cells. The result demonstrated that GFP-FSCN1 was detected in anti-Flag immunoprecipitate from COS-7 cells co-transfected with Flag-*Mage*-*g1* and GFP-*Fscn1* (Fig. 4b). Reciprocal assays showed that Flag-MAGE-G1 was detected in anti-GFP immunoprecipitate from COS-7 cells co-transfected with Flag-*Mage*-*g1* and GFP-*Fscn1* (Fig. 4c). For negative control, there was no GFP-FSCN1 or Flag-MAGE-G1 detected in anti-Flag or anti-GFP antibody immunoprecipitates from cells co-transfected with Flag-*Mage*-*g1* and GFP-*Fscn1*, respectively (Fig. 4b and c).

To further confirm the interaction between MAGE-G1 and FSCN1 during neuronal differentiation, we endogenous immunoprecipitation was performed in P19 cells after treated with RA for 6 days. Results showed that endogenous FSCN1 was co- immunoprecipited with endogenous MAGE-G1 by anti-MAGE-G1 antibody in P19 cells after 6-day treatment with RA s, but not by control IgG (Fig. 4d). The fidelity of anti-MAGE-G1 antibody (B-Bridge, USA) and anti-FSCN1 antibody (Sigma-Aldrich, USA) used in endogenous immunoprecipitation experiments was confirmed (Supplementary Figure 3a and b).

### Validation of the Interaction between MAGE-G1 and VIME

**Figure 5:**
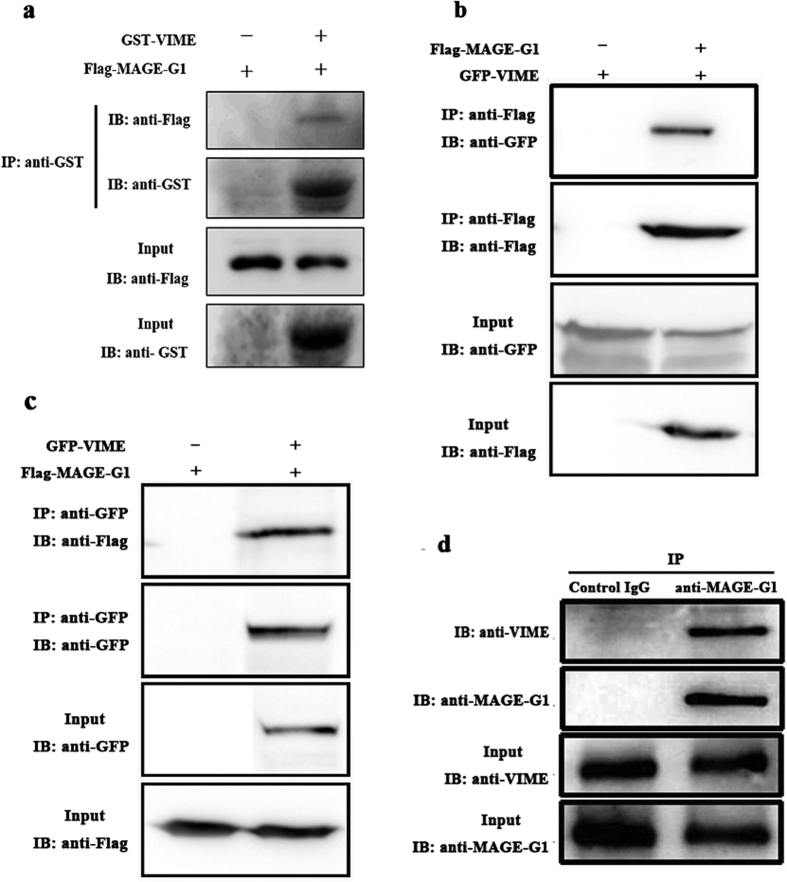
Validation of the interaction between MAGE-G1 and VIME by GST pull-down and co-immunoprecipitation experiments. (**a**) GST or GST-VIME proteins were expressed in *Escherichia coli* BL21 respectively and purified with Glutathione-Sepharose 4B beads and washed, then beads were incubated with Flag-MAGE-G1 expressed in HEK293T. Flag-MAGE-G1 and GST-VIME were detected with indicated antibody. Full-length blots are included in a Supplementary Information. (**b**) COS-7 cells were co-transfected with either Flag-*Mage*-*g1* plus GFP-*Vime* or pCMV-3 × Flag empty vector plus GFP-*Vime* expression plasmids. 25 μg of whole cell protein lysate was used as input to confirm the expression of the Flag-MAGE-G1 (with anti-Flag) or GFP-VIME (with anti-GFP) by immunoblotting (IB). The rest of cell lysates were incubated with anti-Flag-magnetics beads. The immunoprecipitated (IP) protein complex was resolved by SDS-PAGE and probed with antibodies against Flag or GFP. (**c**) COS-7 cells were co-transfected with either Flag-*Mage*-*g1* plus GFP-*Vime* or pEGFP empty vector plus Flag- *Mage*-*g1* expression plasmids. The experiment procedure was same as that mentioned above except that cell lysates were immunoprecipitaed with anti-GFP-magnetics beads. (**d**) Whole cell lysates from RA-treated P19 cells were immunoprecipitated (IP) with anti-MAGE-G1 antibody. IgG antibody was used as negative control of immunoprecipitation and 25 μg whole cell lysate was used as input. The immunoblotting (IB) were probed for the immunoprecipitated proteins with anti-VIME antibody.

To confirm the interaction between MAGE-G1 and VIME, we analyzed the association of these two proteins by GST pull-down assay in bacteria and by immunoprecipitation in transfected mammalian cells. GST or GST-VIME expressed in *Escherichia coli* BL21 were incubated with Flag-MAGE-G1 expressed in HEK293T, and precipitated by Glutathione-Sepharose 4B beads. The result showed that Flag-MAGE-G1 were detected respectively in the GST-VIME precipitate (Fig. 5a). Expression vectors Flag-*Mage*-*g1* and GFP-*Vime* were constructed and transiently co-transfected into COS-7 cells. Empty vectors pCMV-3 × Flag and pGFP-N1 were used as a negative control. Immunoprecipitation and immunoblotting analysis were performed on cell lysates from those transfected cells. The result showed that GFP-VIME was detected in the anti-Flag immunoprecipitate from COS-7 cells co-transfected with Flag-*Mage*-*g1* and GFP-*Vime*, but not detected in COS-7 cells co-transfected with pCMV-3 × Flag and GFP-*Vime* (Fig. 5b). Similarly, Flag-MAGE-G1 was detected in the anti-GFP immunoprecipitate from COS-7 cells co-transfected with Flag-*Mage*-*g1* and GFP-*Vime*, but not in COS-7 cells co-transfected with pGFP-N1 and Flag-*Mage*-*g1* (Fig. 5c).

To analyze the endogenous complex formation *in vivo* between MAGE-G1 and VIME during neuronal differentiation, we carried out endogenous immunoprecipitation in P19 cells after 6-day treatment with RA. The results showed that endogenous VIME was co-immunoprecipited with endogenous MAGE-G1 by anti-MAGE-G1 antibody and no VIME was immunoprecipited in control IgG immunoprecipitate (Fig. 5d). The fidelity of anti-MAGE-G1 antibody (B-Bridge, USA) and anti-VIME antibody (Sigma-Aldrich, USA) used in endogenous immunoprecipitation experiments was confirmed (Supplementary Figure 3a and c).

### The functions of the interaction between MAGE-G1 and FSCN1 or VIME in RA-induced P19 differentiation

**Figure 6:**
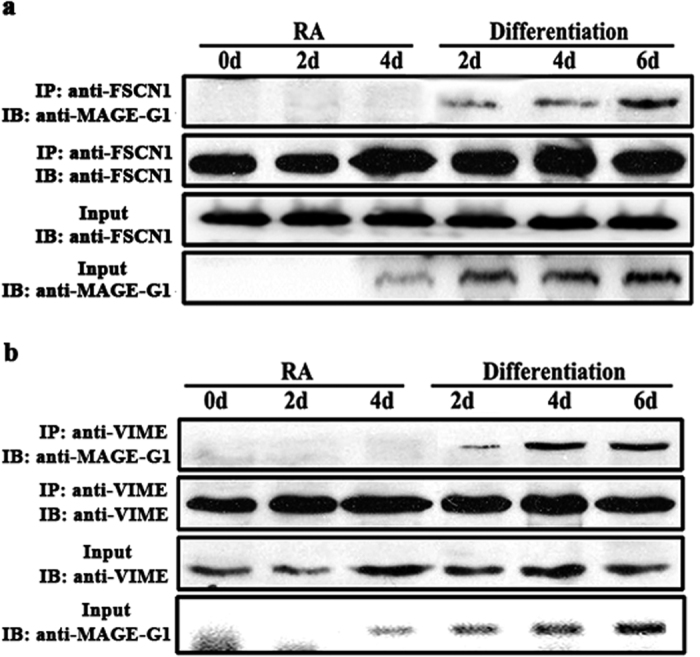
Changes of the interactions between MAGE-G1 and FSCN1 or VIME function in RA-induced P19 neuronal differentiation process. P19 cells were cultured in minimum essential medium containing 1 μM RA for 4 days, and then were replanted in N2 serum-free medium for another 2, 4, 6 days to induce differentiation. Proteins were extracted by IP Lysis Buffer. P19 cells cultured in RA-containing medium for 0, 2, 4 days were used as control. (**a**) Proteins were immunoprecipitated by anti-FSCN1 antibody and the immunoprecipitates were detected by immunoblotting using anti-MAGE-G1 antibody. (**b**) Proteins were immunoprecipitated by anti-VIME antibody and the immunoprecipitates were detected by immunoblotting using anti-MAGE-G1 antibody.

To explore whether the interactions between MAGE-G1 and FSCN1 or VIME function in RA-induced neuronal differentiation process, we performed endogenous co-immunoprecipitation experiments using P19 cells which stayed different stages of RA-induced neuronal differentiation. P19 cells were cultured in minimum essential medium containing 1 μM RA for 4 days, and then were replanted in N2 serum-free medium for another 2, 4, 6 days to induce differentiation. Proteins were extracted by IP Lysis Buffer and immunoprecipitated respectively by related antibody. The immunoprecipitates were detected by immunoblotting. P19 cells cultured in RA-containing medium for 0, 2, 4 days were used as control. The test showed that the expression of MAGE-G1 in P19 cells increased with differentiation time, but the expression of FSCN1 and VIME were constant in P19 cells (Fig. 6). With the increase of MAGE-G1 expression, FSCN1 (Fig. 6a) or VIME (Fig. 6b) detected by co-IP was increased correspondingly. There was no FSCN1 or VIME detected in immunoprecipitate from control groups in which MAGE-G1 did not express or expressed at a low level. Those results showed that the MAGE-G1 expression was increased with the differentiation time and the interactions between MAGE-G1 and FSCN1 or VIME strengthen as well.

In addition, to further explore the functions of the interactions of MAGE-G1 with FSCN1 or VIME, overexpression experiments were performed in differentiating P19 cells. Flag-MAGE-G1 and GFP-FSCN1, Flag-MAGE-G1 and GFP-VIME were respectively overexpressed in P19 cells, and then the expression changes of neural-specific proteins, neuron-specific III β-tubulin and growth-associated protein 43 (GAP43), and apoptosis protein active Caspase 3 were investigated. However, there were no obvious changes in the level of those proteins. These results implied that the interaction of MAGE-G1 with FSCN1 or VIME might not function through these signal molecules in differentiating P19 cells (Supplementary Figure 4). Further studies are required to explore the very functions of the interactions between MAGE-G1 and FSCN1 or VIME in differentiation.

## Discussion

In our previous study, we found that *MAGE*-*G1* mRNA had 5.85-fold increase in P19 cells at 6th day post RA treatment compared with the RA-untreated^16^. It suggested that MAGE-G1 might play a significant role in the early process of neurogenesis. However, the detailed function of MAGE-G1 in neurogenesis remains unclear. To date, only two proteins, E2F1 and p75NTR, have been found interact with MAGE-G1. Previous studies found that MAGE-G1 could reduce cell proliferation, and such effect was mediated by its interaction with the E2F1 transcriptional activator. Interaction of MAGE-G1 with the p75NTR may be involved in brain development^6^. In this study, based on the model of RA-induced neuronal differentiation of P19 cells, SILAC-immunoprecipitation quantitative proteomics was used to identify interaction partners of MAGE-G1. After analysis and validation, FSCN1 and VIME were found to be interacted with MAGE-G1 during RA-induced neuronal differentiation. However, two known MAGE-G1 interactors, E2F1 and p75NTR, were not identified in our experiment. We speculated that our IP lysis buffer was too mild to extract membrane protein p75NTR and nucleoprotein E2F1.

Mammals have three FSCN-coding genes, of which *FSCN2* and *FSCN3* are expressed in narrow domains^17^,^18^, whereas FSCN1 is broadly and dynamically expressed. FSCN-1 is abundant early in development, especially in the central nervous system (CNS) and migrating cells, and then downregulated as cells maturation^19^,^20^,^21^. As a highly conserved actin-binding protein, FSCN1 has diverse roles in the developmental and physiological regulation of cellular morphology and function^22^,^23^,^24^,^25^. In nonneuronal cells, FSCN1 plays numerous roles in the formation of protrusions that regulate adhesion and motility, including tissue invasion by tumor cells^26^,^27^,^28^. FSCN1 is also required for normal brain development by regulating neuronal differentiation^29^. FSCN1 insufficiency or dysregulation might underlie disorders of brain development and plasticity, resulting in intellectual disability^30^.

FSCN1 also binds to noncytoskeletal proteins. FSCN1 is a substrate of protein kinase C alpha (PKCα) *in vitro* and *in vivo*^31^. The phosphorylation of FSCN1 at Ser-39 inhibits its actin-bundling activity and confers an additional activity, binding of the regulatory domain of active PKCα^32^. A third interaction of FSCN1 is with the cytoplasmic domain of the p75NTR^33^. Interestingly, p75NTR could also interact with MAGE-G1^6^, which was identified to be an interactor of FSCN1 in this study. So it was suggested that the interaction between FSCN1, p75NTR and MAGE-G1 may induce a direct effect of neurotrophins on actin cytoskeletal rearrangement changes during RA-induced neuronal differentiation of P19 cells.

VIME is one of the highly conserved proteins of the type III intermediate filament (IF) protein family. During development, VIME expression is predominant in the primitive streak stage, while in adults VIME expression is limited to connective tissue mesenchymal cells, in CNS and in muscle^34^. VIME has been shown to participate in a number of critical functions, often related to organization of proteins that are involved in adhesion, migration, and cell signaling. Recently, many different studies have linked VIME to signal transduction and it has been suggested that they would act as signaling platforms and scaffolds for signaling molecules. VIME can interact with RhoA-binding kinase α (ROKα) that directly phosphorylate VIME^35^. The activation of RhoA resulted in a ROKα-dependent collapse of the VIME network with simultaneous release of ROKα bound to VIME filaments and translocation of ROKα to the cell periphery^35^. VIME networks can also interact with kinases that do not directly phosphorylate VIME. VIME is associated with Raf-1 kinase and activation of the kinase induces VIME phosphorylation^36^. VIME phosphorylation is also controlled by PKCε interacted with VIME on the membrane vesicles^37^. In this study, we found that VIME could be interacted with MAGE-G1 during RA-induced neuronal differentiation of P19 cells, and those findings imply that VIME filaments might work as a binding platform for MAGE-G1 signaling pathway, or its crosstalk with above mentioned VIME-involved signaling pathway during neuronal differentiation.

## Conclusion

In this study, we identified FSCN1 and VIME, which could regulate neuronal differentiation and related to organization of proteins respectively, as two novel interactors of MAGE-G1 during RA-induced neuronal differentiation of P19 cells. Those results imply that the interaction between FSCN1 and MAGE-G1 may induct a direct effect on actin cytoskeletal rearrangement changes during neuronal differentiation. VIME might work as a binding platform for MAGE-G1 signaling pathway, or its crosstalk with VIME-involved signaling pathway. Our findings provide a functional linkage between MAGE-G1 and FSCN1 or VIME and may provide some clues for novel signaling nexuses.

## Methods

### Plasmid Construction

The mouse *Mage*-*g1* cDNA was amplified using primers 5′-ATACTCGAGATGTTGCAGAAGCCGAGG-3′ and 5′-ATAGAATTCAGAGGATGTGGCTGGGG-3′ by RT-PCR from mouse embryonic carcinoma P19 cells cDNA library, and then was subcloned into the Xho I and EcoR I sites of pCMV-3 × Flag vector (Addgene, USA), which is modified to contain COOH-terminal three tandem Flag tags. RFP-MAGE-G1 was constructed by inserting PCR amplified fragment into pDsRed2-N1 vector (Clontech, China). The DNA sequence encoding mouse FSCN1 and VIME protein were amplified by PCR from P19 cell cDNA library. GFP-FSCN1 and GFP-VIME were constructed by inserting PCR amplified fragment respectively into pEGFP-N1 vector (BD Biosciences, USA).

### P19 Cell Culture and Stable Transfection

P19 cells were obtained from the American Type Culture Collection (ATCC, CRL 1825) and cultured in Dulbecco’s modified Eagle’s medium (DMEM) (Invitrogen, USA) supplemented with 10% fetal bovine serum (Invitrogen, USA), 2 mM L-glutamine, 1% penicillin/streptomycin (Sigma-Aldrich, USA) under 5% CO_2_ atmosphere at 37 °C. The Flag-*Mage*-*g1* and empty vector pCMV-3 × Flag plasmids were transfected respectively into the P19 cells by Lipofectamine 2000 (Invitrogen, USA). After 48-hour transfection, the media was replaced with G418-containing (800 μg/ml) medium. Individual colonies were picked after 2-week selection. Transfection efficiency was confirmed by measuring the expression of MAGE-G1 by immunoblotting. Stably transfected cells were maintained in the media with 200 μg/ml G418.

### Stable Isotope Labeling

The P19 cells stably transfected Flag-*Mage*-*g1* plasmid were grown in SILAC DMEM “heavy” media (Thermo, USA) without lysine and arginine, supplemented with 10% dialyzed fetal calf serum (Thermo, USA), 1% penicillin/streptomycin (Sigma-Aldrich,USA), 100 μg/ml L-arginine-HCl and 100 μg/ml ^13^C_6_-L-lysine-2HCl (both from Thermo, USA). Meanwhile, the P19 cells stably transfected empty vector pCMV-3 × Flag plasmid were grown in SILAC DMEM “light” media (Thermo, USA) without lysine and arginine. This two cell populations were grown in corresponding culture medium for at least five cell divisions by changing medium every 2 or 3 days.

### P19 Cell Differentiation

To induce neuronal differentiation, the stably transfected P19 cells were cultured in bacteriological-grade Petri dishes at a seeding density of 1 × 10^5^ cells/ml in the presence of 1 μM all-trans-retinoic acid (Sigma-Aldrich, USA) in minimum essential medium (Wako, Japan) supplemented with 10% fetal bovine serum (Sigma-Aldrich, USA)^38^. After 4-day culture, cells were dissociated into single cell by 0.05% trypsin and were replanted in a poly-L-lysine coated tissue-culture dish at a density of 3 × 10^5^ cells/ml in an N2 serum-free medium (DMEM/F12 supplemented with 5 mg/mL insulin, 50 mg/ml human transferrin, 20 nM progesterone, 60 mM putresine, and 30 nM sodium selenite). The cells were then maintained for at most 6 days with replacement of medium every 2 days.

### Immunoprecipitation Coupled with Liquid Chromatography Tandem Mass Spectrometry (LC-MS/MS)

This two group cells (light and heavy) were harvested and lysed respectively in IP Lysis Buffer (Thermo, USA) (25 mM Tris•HCl pH 7.4, 150 mM NaCl, 1% NP-40, 1 mM EDTA, 5% glycerol) supplemented with protease and phosphatase inhibitors (Roche, Switzerland). After the protein concentration being normalized, equal protein amounts of each cell lysate were mix. The equally mixed sample (2 mg) were incubated with 10 μg rabbit anti-Flag polyclonal antibody (MBL,USA) in 1 ml IP Lysis Buffer overnight at 4 °C, and then were precipitated with 20 μl Protein A/G Plus-agarose (Roche, Switzerland). The immunoprecipitates were separated by 12% SDS–polyacrylamide gel electrophoresis. After being stained with Coomassie Brilliant Blue G-250, each lane of the gel was cut into 10 gel slices (0.5 cm × 0.5 cm) and then analyzed by LC-MS/MS at National Center of Biomedical Analysis (China).

### Database Searching and Criteria for Protein Identification

Tandem mass spectra were extracted, charge state deconvoluted and deisotoped by Mascot Distiller version 2.4.3.3. All MS/MS data were analyzed using Mascot (Matrix Science, London, UK; version 2.4.1). Mascot was set up to search the SwissProt_2013_01 database (selected for Mus., 2013.01, 16638 entries). Detailed information of database searching was previously described^39^. Scaffold (version Scaffold_4.0.5, Proteome Software Inc., Portland, OR) was used to validate MS/MS based peptide and protein identifications. Peptide identifications were accepted if they could be established at greater than 95.0% probability by the Peptide Prophet algorithm with Scaffold delta-mass correction^40^. Protein identifications were accepted if they could be established at greater than 80.0% probability and contained at least 1 identified peptide. Protein probabilities were assigned by the Protein Prophet algorithm^41^. Proteins that contained similar peptides and could not be differentiated on MS/MS analysis alone were grouped to satisfy the principles of parsimony.

### GST pull-down assay

GST, GST-FSCN1 or GST-VIME proteins were expressed in *Escherichia coli* BL21 and purified with Glutathione-Sepharose 4B beads (GE Healthcare, UK) and washed, then beads were incubated with Flag-MAGE-G1 expressed in HEK293T for 4 h at 4 °C. Beads were washed and proteins were eluted, followed by immunoblotting.

### Co-immunoprecipitation and Immunoblotting

Cells were harvested and lysed respectively in IP Lysis Buffer (Thermo, USA) supplemented with protease and phosphatase inhibitors (Roche, Switzerland). The protein samples were incubated with indicated antibody in 1 mL IP Lysis Buffer overnight at 4 °C, and then were precipitated with 20 μL Protein A/G Plus-agarose (Roche, Switzerland). After a brief centrifugation, the pellet was washed 3 times with IP lysis buffer. The lysates and immunoprecipitates were analyzed by immunoblotting.

Immunoblotting was performed using indicated primary antibodies: anti-MAGE-G1 (B-Bridge, USA), anti-GFP (Proteintech, USA), anti-GAP43 (Sigma-Aldrich, USA), anti-Neuron-specific III β-tubulin (Bioworld, China), anti-GAPDH (Bioworld, China), anti-active Caspase3 (Sigma-Aldrich, USA), anti-FSCN1 (Sigma-Aldrich, USA) and anti-VIME (Sigma-Aldrich, USA), anti-GST (Proteintech, USA), anti-Flag (MBL, USA). Detailed information of immunoblotting analysis was previously described^42^.

## Additional Information

**How to cite this article**: Liu, Y. *et al*. Identification of Novel MAGE-G1-Interacting Partners in Retinoic Acid-Induced P19 Neuronal Differentiation Using SILAC-Based Proteomics. *Sci. Rep.* **7**, 44699; doi: 10.1038/srep44699 (2017).

**Publisher's note:** Springer Nature remains neutral with regard to jurisdictional claims in published maps and institutional affiliations.

## Supplementary Material

Supplementary Information

## Figures and Tables

**Table 1:** MAGE-G1-associated proteins with SILAC ratios having significant abundance changes (L/H ratios >1.70 *i.e*. Log_2_ (L/H ratios) >0.7).

Protein name	Accession Number	Molecular Weight	log _2_(Ratio (H/L))
Vimentin	VIME_MOUSE	54 kDa	6.6
Fascin	FSCN1_MOUSE	55 kDa	6.6
Actin-related protein 3	ARP3_MOUSE	47 kDa	5.4
Actin, cytoplasmic 1	ACTB_MOUSE	42 kDa	5.3
Alpha-actinin-4	ACTN4_MOUSE	105 kDa	5.3
Plastin-3	PLST_MOUSE	71 kDa	5.1
Actin, cytoplasmic 2	ACTG_MOUSE	42 kDa	4.6
Drebrin	DREB_MOUSE	77 kDa	4.5
Coronin-1C	COR1C_MOUSE	53 kDa	3.9
Coronin-1B	COR1B_MOUSE	54 kDa	3
Glutathione S-transferase P 1	GSTP1_MOUSE	24 kDa	3
Leucine-rich repeat flightless-interacting protein 2	LRRF2_MOUSE	47 kDa	2.4
DNA ligase 3	DNLI3_MOUSE	113 kDa	2.2
Actin, alpha cardiac muscle 1	ACTC_MOUSE	42 kDa	2.2
Histone H1.1	H11_MOUSE	22 kDa	2.2
Tropomodulin-3	TMOD3_MOUSE	40 kDa	1.9
Tubulin beta-5 chain	TBB5_MOUSE	50 kDa	1.6
Inosine-5′-monophosphate dehydrogenase 2	IMDH2_MOUSE	56 kDa	1.5
Paraspeckle component 1	PSPC1_MOUSE	59 kDa	1.3
Myosin-10	MYH10_MOUSE	229 kDa	1.1
Heterogeneous nuclear ribonucleoprotein F	HNRPF_MOUSE	46 kDa	1.1
Tubulin alpha-1C chain	TBA1C_MOUSE	50 kDa	1
Insulin-like growth factor 2 mRNA-binding protein 1	IF2B1_MOUSE	63 kDa	1
Tubulin beta-4B chain	TBB4B_MOUSE	50 kDa	1
60S ribosomal protein L4	RL4_MOUSE	47 kDa	0.9
Poly [ADP-ribose] polymerase 1	PARP1_MOUSE	113 kDa	0.9
Guanine nucleotide-binding protein subunit beta-2-like 1	GBLP_MOUSE	35 kDa	0.9
