# Supplementary material for: Identification of Novel MAGE-G1-Interacting Partners in Retinoic Acid-Induced P19 Neuronal Differentiation Using SILAC-Based Proteomics
Source: Sci Rep. 2017 Apr 4;7:44699. doi: 10.1038/srep44699 (PMC5379670; doi:10.1038/srep44699)
Supplement: Supplementary Information [file srep44699-s1.pdf]

Supplementary Information

Identification of Novel MAGE-G1-Interacting Partners in Retinoic Acid-Induced P19 Neuronal Differentiation Using SILAC-Based Proteomics

Yong Liu*[+], Yujian Chen[+], Shide Lin[+], Shuguang Yang, Shaojun Liu*

*corresponding authors: Yong Liu, liuyongxiao1225@hotmail.com; Shaojun Liu, liusj@bmi.ac.cn.

[+]these authors contributed equally to this work.

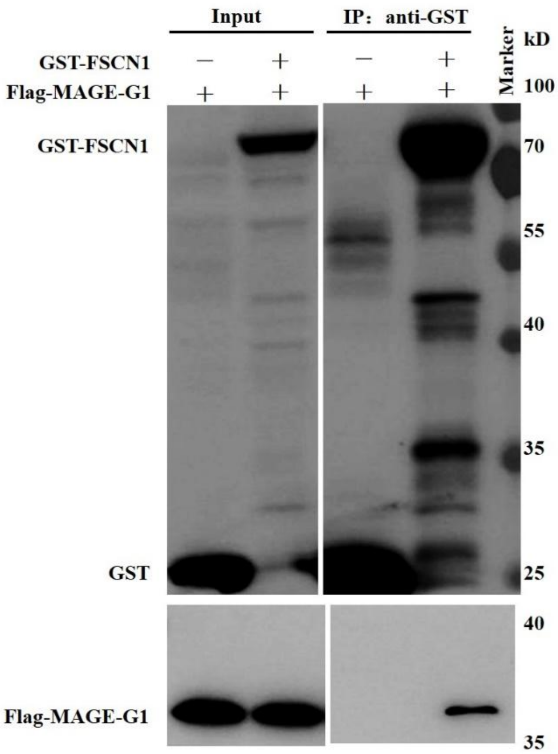

Supplementary Figure 1. Full-length blot figures of validation of the interaction between MAGE-G1 and FSCN1 by GST pull-down assay. GST or GST-FSCN1 proteins were expressed in *Escherichia coli* BL21 respectively and purified with Glutathione-Sepharose 4B beads and washed, then beads were incubated with Flag-MAGE-G1 expressed in HEK293T. Flag-MAGE-G1 and GST-FSCN1 were detected with anti-GST and anti-Flag antibody.

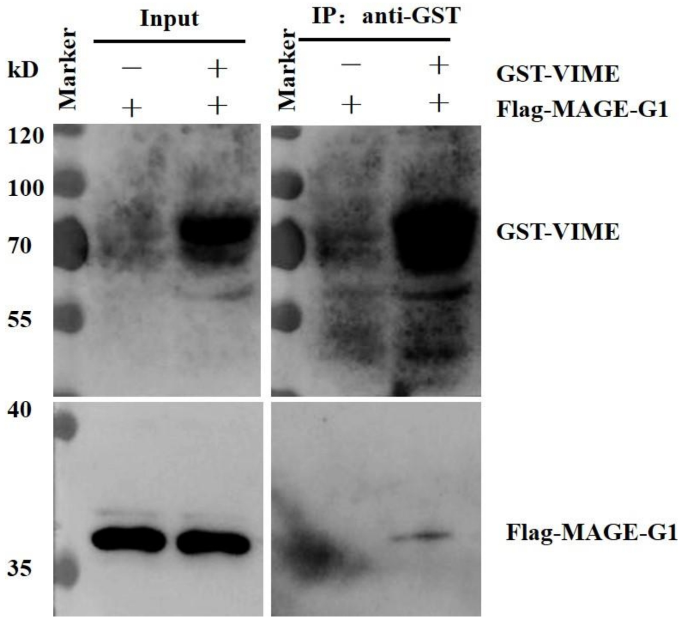

Supplementary Figure 2. Full-length blot figures of validation of the interaction between MAGE-G1 and VIME by GST pull-down assay. (a) GST or GST-VIME proteins were expressed in *Escherichia coli* BL21 respectively and purified with Glutathione-Sepharose 4B beads and washed, then beads were incubated with Flag-MAGE-G1 expressed in HEK293T. Flag-MAGE-G1 and GST-VIME were detected with anti-GST and anti-Flag antibody.

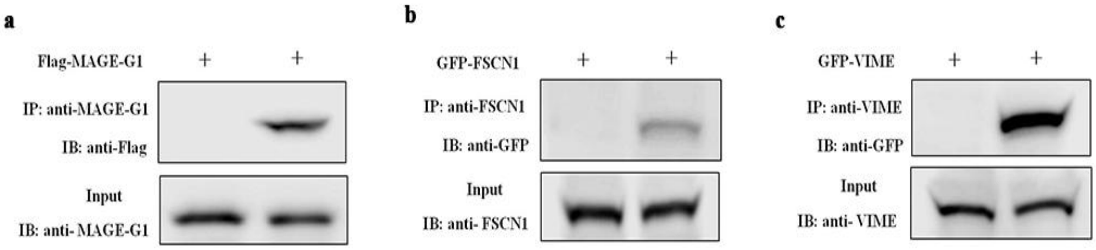

Supplementary Figure 3. Confirm the fidelity and validation of the antibodies used in endogenous immunoprecipitation experiments. To confirm the fidelities of anti-MAGE-G1 antibody (B-Bridge, USA), anti-FSCN1 antibody (Sigma-Aldrich, USA) and anti-VIME antibody (Sigma-Aldrich, USA) used in endogenous immunoprecipitation experiments, Flag-MAGE-G1, GFP-FSCN1 and GFP-VIME proteins expressed respectively in HEK293T cells were extracted and immunoprecipitated by related antibody**.** Then the immunoprecipitates were detected by immunoblotting using anti-Flag antibody (MBL, USA) and anti-GFP antibody (Proteintech, USA). Our results showed that Flag-MAGE-G1, GFP-FSCN1 and GFP-VIME were detected respectively in anti- MAGE-G1, anti-FSCN1 and anti-VIME immunoprecipitates by anti-Flag antibody and anti-GFP antibody. **(a)** Flag-MAGE-G1 protein expressed in HEK293T cells was extracted and immunoprecipitated by anti-MAGE-G1 antibody (B-Bridge, USA). The immunoprecipitates were detected by immunoblotting using anti-Flag antibody (MBL, USA). **(b)** GFP-FSCN1 protein

expressed in HEK293T cells was extracted and immunoprecipitated by anti-FSCN1 antibody (Sigma-Aldrich, USA). The immunoprecipitates were detected by immunoblotting using anti-GFP antibody (Proteintech, USA). **(c)** GFP-VIME protein expressed in HEK293T cells was extracted and immunoprecipitated by anti-VIME antibody (Sigma-Aldrich, USA). The immunoprecipitates were detected by immunoblotting using anti-GFP antibody (Proteintech, USA). IgG antibody was used as a negative control in immunoprecipitation.

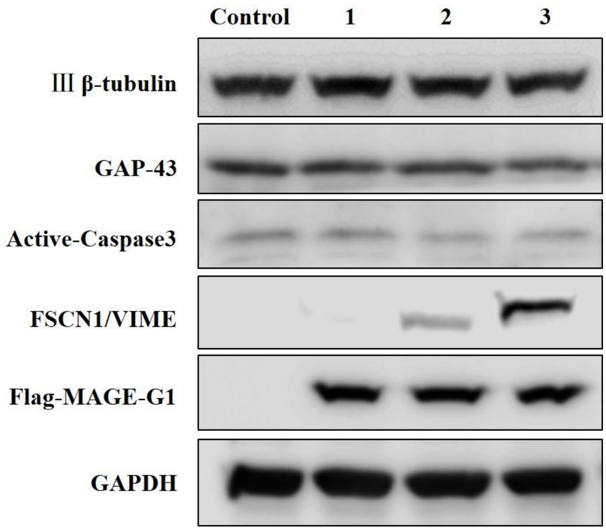

Figure 4. The expression changes of related proteins in P19 differentiation that overexpressed MAGE-G1 and FSCN1 or VIME. *Control*, P19 cells that stably expressed Flag and GFP. *1*, P19 cells that stably expressed Flag and GFP. P19 cells that stably expressed Flag-MAGE-G1 and GFP. *2*, P19 cells that stably expressed Flag-MAGE-G1 and GFP-FSCN1. *3*, P19 cells that stably expressed Flag-MAGE-G1 and GFP-VIME1.

Supplementary Table 1. A total of 57 proteins were quantified with SILAC assay.

Protein name	Accession Number	Molecular Weight	$\log_2$ (Ratio (H/L))
Vimentin	VIME_MOUSE	54 kDa	6.6
Fascin	FSCN1_MOUSE	55 kDa	6.6
Actin-related protein 3	ARP3_MOUSE	47 kDa	5.4
Actin, cytoplasmic 1	ACTB_MOUSE	42 kDa	5.3
Alpha-actinin-4	ACTN4_MOUSE	105 kDa	5.3
Plastin-3	PLST_MOUSE	71 kDa	5.1
Actin, cytoplasmic 2	ACTG_MOUSE	42 kDa	4.6
Drebrin	DREB_MOUSE	77 kDa	4.5
Coronin-1C	COR1C_MOUSE	53 kDa	3.9
Coronin-1B	COR1B_MOUSE	54 kDa	3
Glutathione S-transferase P 1	GSTP1_MOUSE	24 kDa	3
Leucine-rich repeat	LRRF2_MOUSE	47 kDa	2.4

flightless-interacting protein 2			
DNA ligase 3	DNLI3_MOUSE	113 kDa	2.2
Actin, alpha cardiac muscle 1	ACTC_MOUSE	42 kDa	2.2
Histone H1.1	H11_MOUSE	22 kDa	2.2
Tropomodulin-3	TMOD3_MOUSE	40 kDa	1.9
Tubulin beta-5 chain	TBB5_MOUSE	50 kDa	1.6
Inosine-5'-monophosphate dehydrogenase 2	IMDH2_MOUSE	56 kDa	1.5
Paraspeckle component 1	PSPC1_MOUSE	59 kDa	1.3
Myosin-10	MYH10_MOUSE	229 kDa	1.1
Heterogeneous nuclear ribonucleoprotein F	HNRPF_MOUSE	46 kDa	1.1
Tubulin alpha-1C chain	TBA1C_MOUSE	50 kDa	1
Insulin-like growth factor 2 mRNA-binding protein 1	IF2B1_MOUSE	63 kDa	1
Tubulin beta-4B chain	TBB4B_MOUSE	50 kDa	1
60S ribosomal protein L4	RL4_MOUSE	47 kDa	0.9
Poly [ADP-ribose] polymerase 1	PARP1_MOUSE	113 kDa	0.9
Guanine nucleotide-binding protein subunit beta-2-like 1	GBLP_MOUSE	35 kDa	0.9
Myosin-9	MYH9_MOUSE	226 kDa	0.7
Eukaryotic initiation factor 4A-I	IF4A1_MOUSE	46 kDa	0.6
Probable ATP-dependent RNA helicase DDX17	DDX17_MOUSE	72 kDa	0.6
Non-POU domain-containing octamer-binding protein	NONO_MOUSE	55 kDa	0.5
Heat shock cognate 71 kDa protein	HSP7C_MOUSE	71 kDa	0.3
Insulin-like growth factor 2 mRNA-binding protein 3	IF2B3_MOUSE	64 kDa	0.3
40S ribosomal protein S3	RS3_MOUSE	27 kDa	0.3
40S ribosomal protein S8	RS8_MOUSE	24 kDa	0.2
Polyadenylate-binding protein 1	PABP1_MOUSE	71 kDa	0.1
Splicing factor, proline- and glutamine-rich	SFPQ_MOUSE	75 kDa	0.1
Spectrin alpha chain, non-erythrocytic 1	SPTN1_MOUSE	285 kDa	0
Ras GTPase-activating protein-binding protein 1	G3BP1_MOUSE	52 kDa	0
60S ribosomal protein L10a	RL10A_MOUSE	25 kDa	0
Spectrin beta chain, non-erythrocytic 1	SPTB2_MOUSE	274 kDa	0
60S ribosomal protein L18	RL18_MOUSE	22 kDa	0
Eukaryotic translation initiation	EIF3D_MOUSE	64 kDa	0

factor 3 subunit D			
GTP-binding nuclear protein Ran	RAN_MOUSE	24 kDa	0
Stress-70 protein, mitochondrial	GRP75_MOUSE	73 kDa	-0.1
60S ribosomal protein L7a	RL7A_MOUSE	30 kDa	-0.1
Unconventional myosin-Ib	MYO1B_MOUSE	129 kDa	-0.2
Elongation factor 1-alpha 1	EF1A1_MOUSE	50 kDa	-0.3
Heat shock protein HSP 90-beta	HS90B_MOUSE	83 kDa	-0.3
Protein flightless-1 homolog	FLII_MOUSE	145 kDa	-0.4
Unconventional myosin-Va	MYO5A_MOUSE	216 kDa	-0.6
T-complex protein 1 subunit alpha	TCPA_MOUSE	60 kDa	-0.7
Glyceraldehyde-3-phosphate dehydrogenase	G3P_MOUSE	36 kDa	-0.9
T-complex protein 1 subunit beta	TCPB_MOUSE	57 kDa	-1
Asparagine--tRNA ligase, cytoplasmic	SYNC_MOUSE	64 kDa	-1
Nucleolin	NUCL_MOUSE	77 kDa	-1.1
T-complex protein 1 subunit gamma	TCPG_MOUSE	61 kDa	-2.7
